# MALDI mass spectrometry imaging of erlotinib administered in combination with bevacizumab in xenograft mice bearing B901L, EGFR-mutated NSCLC cells

**DOI:** 10.1038/s41598-017-17211-6

**Published:** 2017-12-01

**Authors:** Masanobu Nishidate, Kaname Yamamoto, Chinami Masuda, Hiroaki Aikawa, Mitsuhiro Hayashi, Takehiko Kawanishi, Akinobu Hamada

**Affiliations:** 1 Translational Clinical Research Science & Strategy Dept., Chugai Pharmaceutical Co., Ltd., 200 Kajiwara, Kamakura, Kanagawa 247-8530 Japan; 2 Product Research Dept., Chugai Pharmaceutical Co., Ltd., 200 Kajiwara, Kamakura, Kanagawa 247-8530 Japan; 3 Division of Clinical Pharmacology and Translational Research, Exploratory Oncology Research and Clinical Trial Center, National Cancer Center, 5-1-1 Tsukiji, Chuo-ku, Tokyo, 104-0045 Japan; 4 Department of Molecular Pharmacology, National Cancer Center Research Institute, 5-1-1 Tsukiji, Chuo-ku, Tokyo, 104-0045 Japan; 5 Department of Medical Oncology and Translational Research, Graduate school of Medical Sciences, Kumamoto University, 1-1-1 Honjo, Chuo-ku, Kumamoto, 860-8556 Japan

## Abstract

Combination therapy of erlotinib plus bevacizumab improves progression-free survival of patients with epidermal growth factor receptor–mutated (EGFR-mutated) advanced non–small-cell lung cancer (NSCLC) compared with erlotinib alone. Although improved delivery and distribution of erlotinib to tumours as a result of the normalization of microvessels by bevacizumab is thought to be one of the underlying mechanisms, there is insufficient supporting evidence. B901L cells derived from EGFR-mutated NSCLC were subcutaneously implanted into mice, and mice were treated with bevacizumab or human IgG followed by treatment with erlotinib. The distribution of erlotinib in their tumours at different times after erlotinib administration was analysed by matrix-assisted laser desorption/ionization mass spectrometry imaging (MALDI MSI). We also analysed the distribution of erlotinib metabolites and the distribution of erlotinib in tumours refractory to erlotinib, which were established by long-term treatment with erlotinib. We found that erlotinib was broadly diffused in the tumours from B901L-implanted xenograft mice, independently of bevacizumab treatment. We also found that erlotinib metabolites were co-localized with erlotinib and that erlotinib in erlotinib-refractory tumours was broadly distributed throughout the tumour tissue. Multivariate imaging approaches using MALDI MSI as applied in this study are of great value for pharmacokinetic studies in drug development.

## Introduction

Erlotinib in combination with bevacizumab was shown in the JO25567 study to improve progression-free survival (PFS) in patients with epidermal growth factor receptor–mutated (EGFR-mutated) advanced non–small-cell lung cancer (NSCLC) compared with treatment with erlotinib alone^1^. Erlotinib is a tyrosine kinase inhibitor targeting EGFR and is effective in treating patients with EGFR-mutated NSCLC^2–4^. Bevacizumab is a humanized anti-VEGF monoclonal antibody, and therapy with bevacizumab in combination with erlotinib has been studied for the treatment of lung cancer^5,6^.

Several mechanisms have been proposed to explain this efficacy. One proposed mechanism is that normalization of microvessels in tumours by bevacizumab results in improvement of drug delivery to tumours^7–9^. Because the interstitial pressure in tumours is higher than that in normal tissue and because the structures of microvessels are immature, appropriate drug delivery and distribution into tumours might be prevented^10,11^. Bevacizumab treatment is known to normalize interstitial pressure and microvessel structure in tumours^12,13^, and as a result, drug delivery to and distribution within tumours are thought to be improved. Drug delivery and distribution are related to efficacy and to adverse events^14,15^; to exert their efficacy, drugs need to be delivered to and distributed within the target tissues. Information on delivery and distribution can also be a great help for selection of new drug candidates. Therefore, a clear understanding of drug delivery and distribution is very important.

Matrix-assisted laser desorption/ionization mass spectrometry imaging (MALDI MSI) is a technique becoming increasingly prominent in pharmacokinetics studies^16–21^. It can acquire data on the presence of analytes together with information on their spatial distributions within a sample, information which is lost with liquid chromatography–tandem mass spectrometry (LC-MS/MS) assays using sample homogenates. Furthermore, unlike with imaging techniques using labelled drugs, MSI can distinguish between drugs and their metabolites on the basis of their mass-to-charge ratios. Therefore, MSI is a promising tool to complement autoradiography which is currently widely used for drug distribution analysis^16^.

With respect to the delivery and distribution of erlotinib in EGFR-mutated NSCLC, several studies have shown important aspects of the relationship between microvessel normalization by antiangiogenic agents and drug delivery and distribution. Quantitation of erlotinib in tumours by LC-MS/MS revealed that treatment with PTK787 (an inhibitor of VEGFR tyrosine kinase, PDGFRβ tyrosine kinase, and c-Kit) improved delivery of erlotinib to tumours derived from a lung cancer cell line^22^, and use of MALDI MSI revealed that inhibition of angiogenesis by bevacizumab improved paclitaxel distribution in tumours derived from an ovarian cancer cell line^23^. However, there is currently insufficient data to conclude that combination therapy with bevacizumab improves delivery and distribution of erlotinib in patients with EGFR-mutated NSCLC. In the former study, information on erlotinib distribution within the tumours was not investigated because sample homogenates were measured using LC-MS/MS. Furthermore, since PTK787 is a multi-kinase inhibitor, it might have activity different from that of bevacizumab which specifically binds to VEGF only. In the latter study, although the distribution of paclitaxel was shown by using MALDI MSI, it cannot be said that erlotinib in lung cancer will show the same distribution as that of paclitaxel in ovarian cancer, since drug distribution depends greatly on the tumour microenvironment and the physicochemical properties of the drug. Moreover, unlike with a cytotoxic drug such as paclitaxel, time-dependent distribution due to interactions with EGFR might also need to be considered.

The aim of this study was to reveal the effect of bevacizumab on the distribution of erlotinib in EGFR-mutated NSCLC by using MALDI MSI. The distribution of erlotinib in tumours from xenograft mice subcutaneously implanted with B901L, an EGFR-mutated NSCLC cell line, and treated or not treated with bevacizumab, was analysed at various time points. In addition, erlotinib distribution in erlotinib-refractory tumours, created by long-term treatment with erlotinib, was also analysed to more fully understand erlotinib distribution.

## Results

### Microvessel density analysis

**Figure 1:**
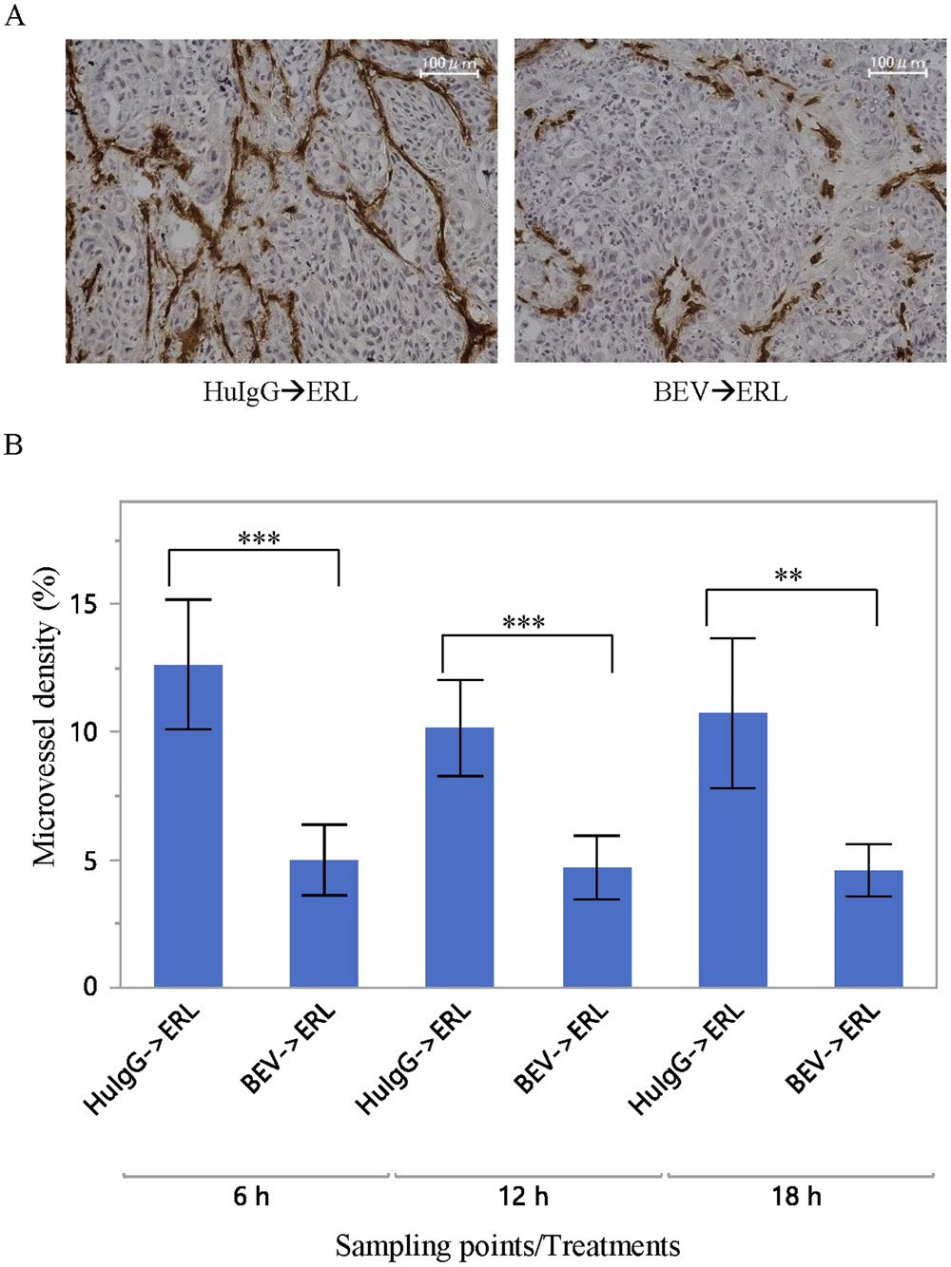
Microvessel density analysis by CD31 staining. (**A**) Representative images of microvessels from tumours treated with erlotinib (ERL) following human IgG (HuIgG, left) or bevacizumab (BEV, right). (**B**) Microvessel density (%) calculated by dividing the CD31-positive area by viable cell area. Mean ± SD are shown (*n* = 5 for each sampling point in each treatment group). Area of microvessels was significantly decreased in tumours from xenograft mice treated with bevacizumab (**P < 0.01 and ***P < 0.001, by Student’s t-test).

Tumours from mice treated with a single dose of erlotinib (60 mg/kg) 5 days after a single dose of bevacizumab (5 mg/kg) or 5 days after a single dose of human IgG (5 mg/kg) (*n* = 5 for each time point in each treatment group) (Table 1) were sectioned and stained with mouse CD31 to evaluate microvessel density. The microvessel density in tumours treated with erlotinib following bevacizumab was significantly lower than that in tumours treated with erlotinib following human IgG (Fig. 1).

**Table 1:** Treatment schemes and collected samples.

Experiment	Treatments	Treatment frequency	Sample collection after last erlotinib administration	*n*
1	Erlotinib (60 mg/kg) 5 days after bevacizumab (5 mg/kg)	Single dose	6 h, 12 h, 18 h	5 (at each time point)
	Erlotinib (60 mg/kg) 5 days after Human IgG (5 mg/kg)	Single dose	6 h, 12 h, 18 h	5 (at each time point)
2	Erlotinib (60 mg/kg)	Daily for 2 days	3 h	5
	Erlotinib (60 mg/kg)	Daily for 37 days	3 h	5

### Pharmacokinetics of erlotinib in tumours and serum

**Figure 2:**
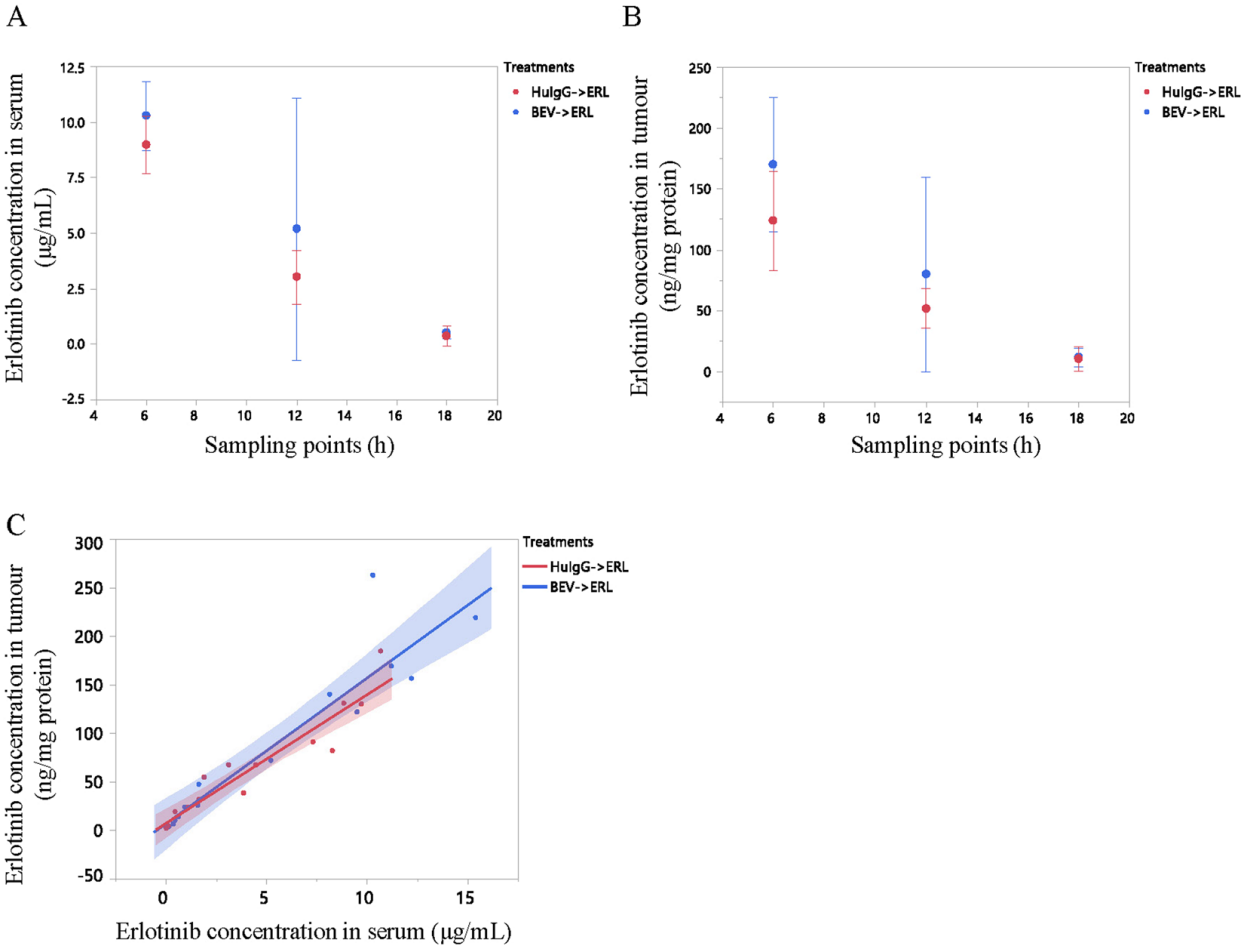
Erlotinib (ERL) concentrations in serum and tumours in mice treated with erlotinib (ERL) following human IgG (HuIgG) or bevacizumab (BEV). Samples were collected at 6 h, 12 h, or 18 h after erlotinib treatment. Erlotinib concentrations in serum (**A**) and tumours (**B**) were measured using LC-MS/MS. Red plots indicate human IgG+ erlotinib treatment and blue plots indicate bevacizumab + erlotinib treatment. Mean ± SD are shown. (**C**) Correlation between serum concentration and tumour concentration of erlotinib for each treatment group; 95% confidence intervals for the two groups overlapped (shaded areas).

Erlotinib concentrations in serum and tumours collected from mice treated with erlotinib (60 mg/kg) following bevacizumab (5 mg/kg) or human IgG (5 mg/kg) at 6 h, 12 h, and 18 h after erlotinib administration (*n* = 5 for each time point in each treatment group, Table 1) were measured by LC-MS/MS. The serum concentrations for each treatment group at each time point were not significantly different (Fig. 2A). The concentrations in tumour tissue for each treatment group at each time point also showed no significant difference (Fig. 2B). The 95% confidence interval of the relationship between serum concentration and tumour concentration in the bevacizumab treatment group overlapped that of the human IgG treatment group (Fig. 2C).

Delivery and distribution of erlotinib in tumours refractory to erlotinib was also analysed. The erlotinib-refractory tumour model was established by long-term erlotinib treatment. Erlotinib concentrations in serum and tumours collected from mice treated with erlotinib (60 mg/kg daily) for 2 days (short term/sensitive) or 37 days (long term/refractory) at 3 h after the last administration (Table 1) were measured by LC-MS/MS (*n* = 5 for each treatment group). Concentrations in serum and tumours were not significantly different between the treatment groups (*P* < 0.05, by Student’s t-test) (Supplementary Fig. S1).

### Visualization of erlotinib distribution in tumours

Measurement of erlotinib in tissue by LC-MS/MS is a very effective approach to understand the delivery of erlotinib to tumours. However, this technique lacks spatial information. To complement the LC-MS/MS analysis, tumour tissues were analysed by MALDI MSI, which could determine both the existence of analytes and their localizations.

To demonstrate that the analytical methods were specific to erlotinib and its metabolites, tumours from mice that received no treatment were analysed by using 2 different MALDI–MSI systems, iMScope (Shimadzu, Kyoto, Japan) and Q Exactive (Thermo Fisher Scientific, Waltham, MA, USA), with AP-SMALDI (TransMIT, Giessen, Germany) as the ion source. Since iMScope was relatively sensitive and produced reproducible intensities compared to Q Exactive, the instrument was mainly used. On the other hands, Q Exactive was used for the analysis of M14 and M13, because of its mass resolving power to separate specific spectra of M14 and M13 from spectra of background noise. No signals indicating erlotinib or its metabolites (M14 or M13) were observed in these tissue sections (Supplementary Fig. S3), and thus the methods developed here could analyse the presence and location of erlotinib and its metabolites specifically.

**Figure 3:**
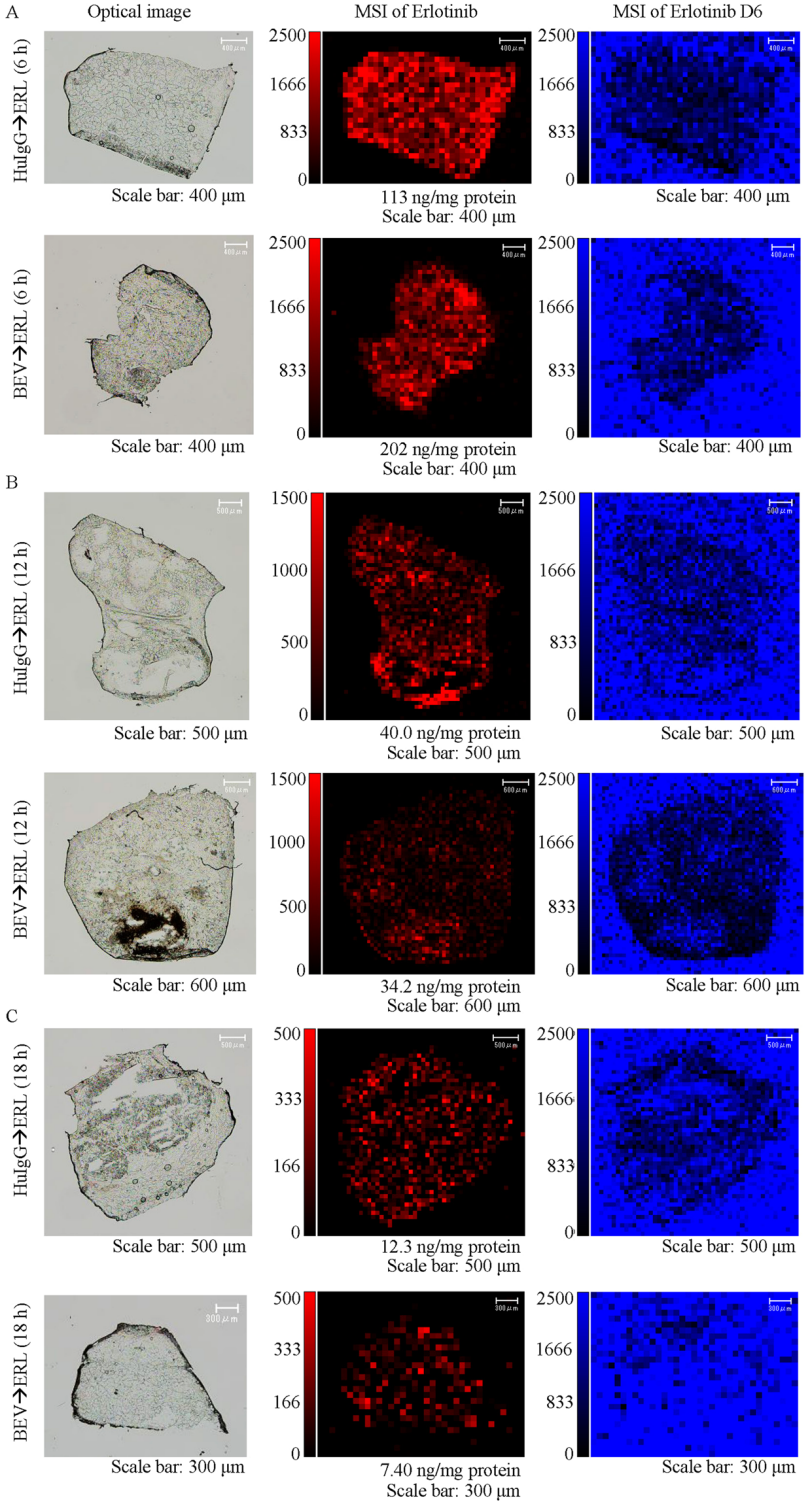
Erlotinib distribution in tumours from mice treated with erlotinib (ERL) following human IgG (HuIgG, upper panels) or bevacizumab (BEV, lower panels). Tumours were collected at 6 h (**A**), 12 h (**B**), or 18 h (**C**) after treatment with erlotinib. The amount of erlotinib measured in an adjacent tissue section is indicated below the erlotinib imaging (centre panels). Erlotinib D6 deposited together with the matrix was also imaged to grasp the trends of ionization in the tissues. Data was obtained using iMScope with 80 μm spatial resolution. The maximum value on the intensity scale was adjusted for each time point to compare between treatment groups. Erlotinib signals are indicated by red and erlotinib D6 intensity is indicated by blue.

Mice treated with erlotinib (60 mg/kg) following bevacizumab (5 mg/kg) or human IgG (5 mg/kg) were killed at 6 h (*n* = 3 in each treatment group), 12 h (*n* = 3 in each treatment group), or 18 h (*n* = 5 in each treatment group) after erlotinib treatment, and their tumours were analysed using iMScope. Erlotinib was broadly distributed in the tumours in both treatment groups at all time points (Fig. 3). Little difference in erlotinib distribution was observed between the treatment groups.

**Figure 4:**
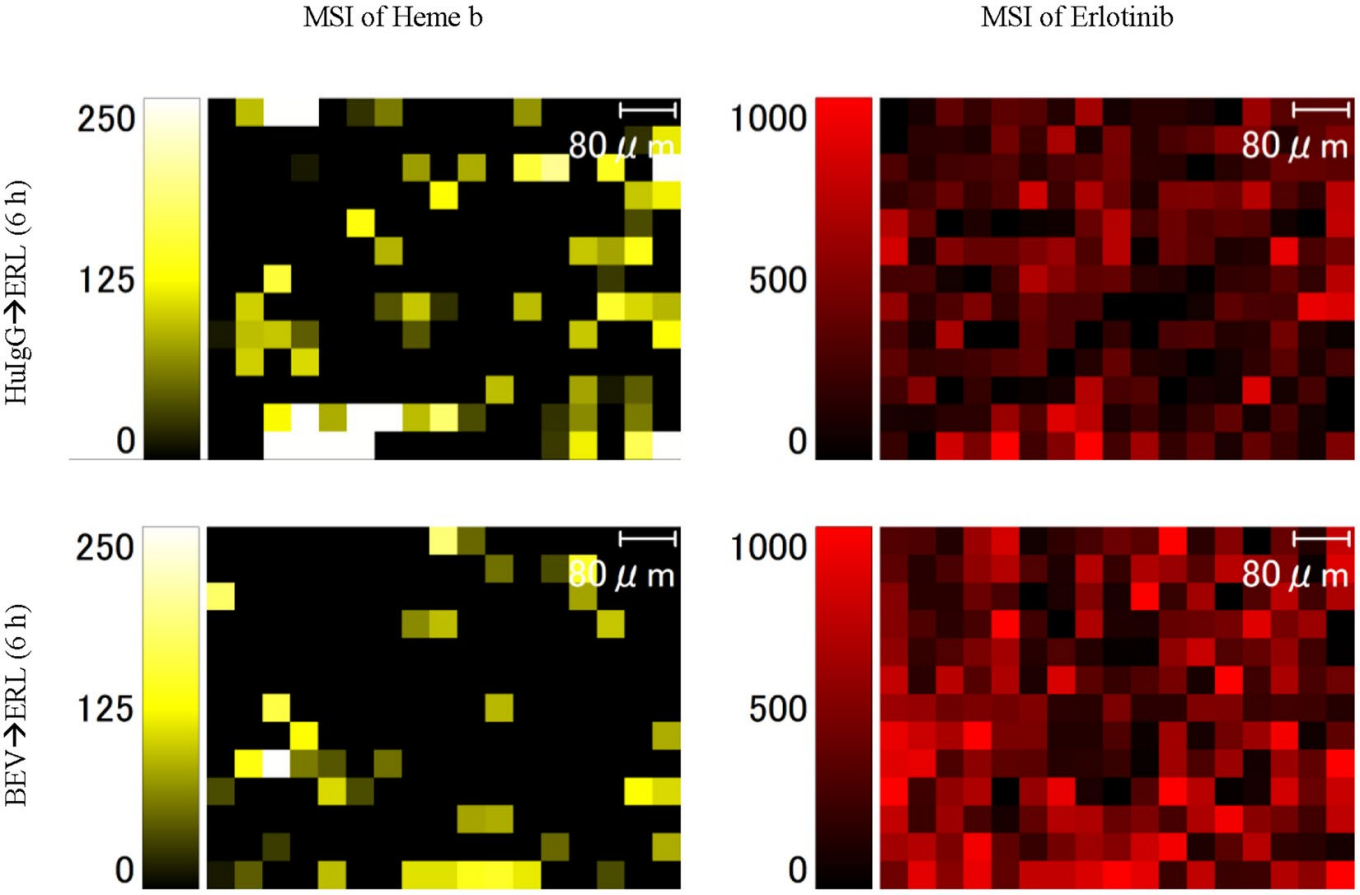
Distribution of heme b and erlotinib in tumours treated with erlotinib (ERL) following human IgG (HuIgG, upper panels) or bevacizumab (BEV, lower panels) in the same areas of tumour tissue. Tumours were collected at 6 h after treatment with erlotinib. The maximum value on the intensity scale was adjusted for each image.

However, it is possible that the observed erlotinib distribution might just reflect blood distribution. To unequivocally demonstrate erlotinib penetration into tumour cells, it is very important to show the correlation between blood distribution and erlotinib distribution in viable tumours. To visualize heme b and erlotinib distribution in the same tumour area, tumours from mice treated with erlotinib (60 mg/kg) following bevacizumab (5 mg/kg) or human IgG (5 mg/kg) were collected at 6 h after erlotinib treatment (*n* = 1 in each treatment group) and the viable tumour areas were analysed by using iMScope at a spatial resolution of 40 µm. Erlotinib could be detected in areas where there was no heme b signal (Fig. 4).

**Figure 5:**
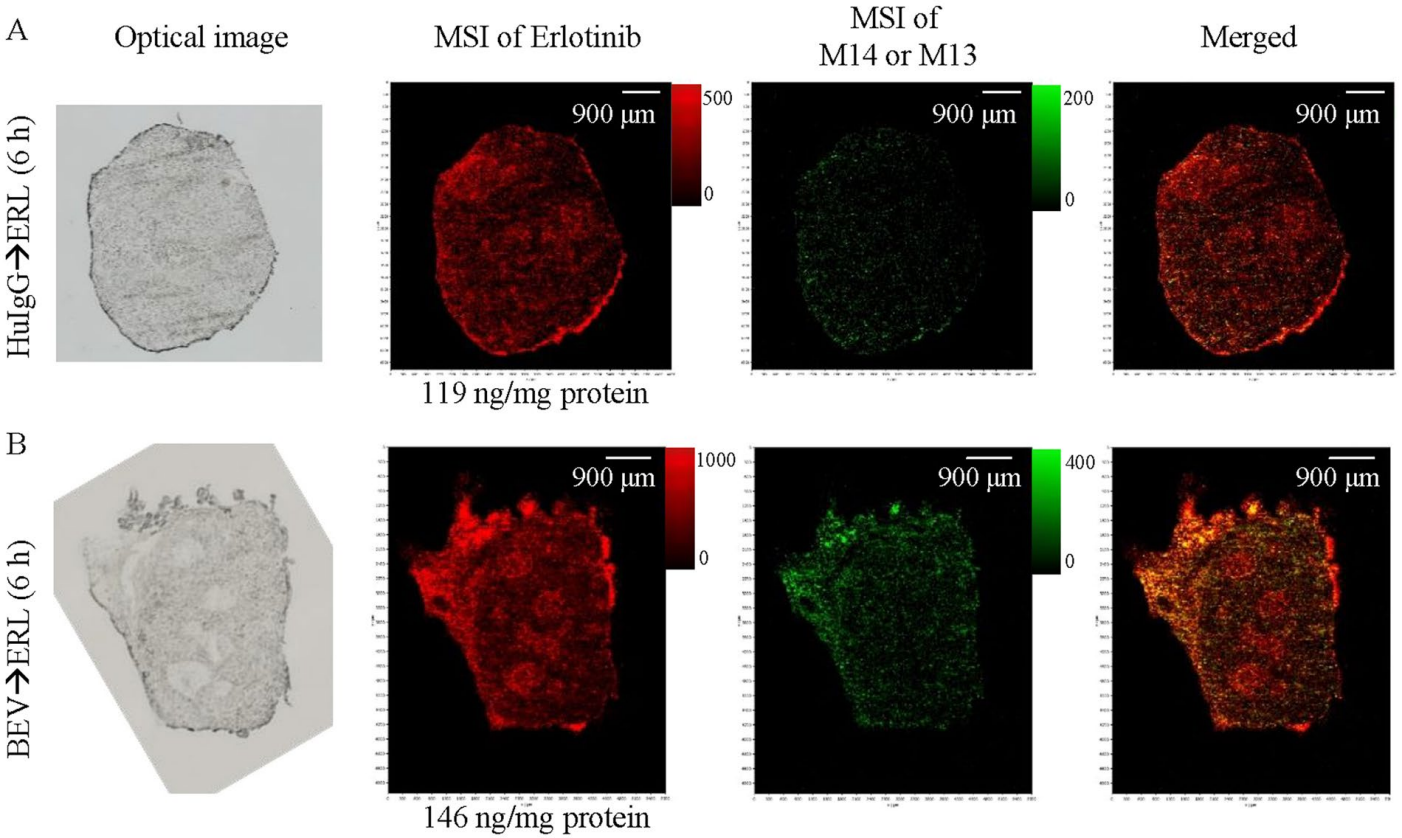
Erlotinib distribution in tumours treated with erlotinib (ERL) following human IgG (HuIgG, **A**) or bevacizumab (BEV, **B**). Tumours were collected at 6 h after treatment with erlotinib. Optical images were taken just before MSI, and are the same tissue sections as the imaged sections. The concentration of erlotinib in an adjacent tissue section is indicated below the erlotinib imaging. The maximum value on the intensity scale was adjusted for each image. Erlotinib signals are indicated in red and signals of its metabolites (M14 or M13) are indicated in green. Right panels show both images merged (orange).

Since erlotinib has an active metabolite, M14 (desmethyl-erlotinib), the distribution of this metabolite should also be analysed. To analyse erlotinib and M14 distribution simultaneously, Q Exactive with AP-SMALDI was used. M14 cannot be distinguished from M13 by this method because it has exactly the same mass. Hence, the spectra acquired for metabolites were the sum of M14 and M13. Tumour tissue with relatively high erlotinib concentrations was selected from mice treated with erlotinib (60 mg/kg) following bevacizumab (5 mg/kg) or human IgG (5 mg/kg) and sacrificed at 6 h (*n* = 1 in each treatment group), because the concentration of metabolites was assumed to be lower than that of erlotinib in such tissue. Measurements using Q Exactive showed that erlotinib and its active metabolites were spread broadly throughout tumour tissue (Fig. 5).

Finally, erlotinib distribution in tumours collected from mice treated with erlotinib (60 mg/kg) daily for 2 days (short term) or 37 days (long term) at 3 h after the last treatment (Table 1) was also analysed using iMScope (*n* = 2 in each treatment group). Erlotinib was diffused throughout the tissue, even in erlotinib-refractory tumours (Supplementary Fig. S4).

## Discussion

In this study we found that the distribution of erlotinib in tumours excised from B901L-implanted xenograft mice was broadly diffused, independently of bevacizumab treatment. We analysed not only the time course of erlotinib in tumours from mice treated with bevacizumab followed by erlotinib but we also examined erlotinib metabolites (M14 and M13). In addition, we also analysed erlotinib-refractory tumours from mice subjected to long-term repeated erlotinib treatment. LC-MS/MS in combination with MSI analysis was more appropriate for investigating erlotinib distribution in tumours than was LC-MS/MS alone.

Erlotinib distribution was not affected by the reduction in microvessel density and was distributed throughout the tissues at each of the measured time points. The metabolites were co-localized with erlotinib, and erlotinib was distributed broadly to the erlotinib-refractory tumours repeatedly treated with erlotinib for 37 days.

M14 is an active metabolite of erlotinib^24^. Since this metabolite has different physicochemical properties (molecular weight, shape, charge, aqueous solubility, etc.) to erlotinib, we decided that it was best to also analyse the distribution of this metabolite too. The observed metabolite spectrum at *m*/*z* 380.1605 also includes results derived from M13, a metabolite in which the other side chain of erlotinib is demethylated (Supplementary Fig. S2A) and which cannot be separated from M14 by only mass spectrometry because of their identical masses. We concluded that since erlotinib metabolites were co-localized with erlotinib, analysing erlotinib distribution alone would be sufficient for this study.

A study using an B901L xenograft model reported that tumours repeatedly treated with erlotinib acquired resistance to the therapy within about a month^25^. Several mechanisms of erlotinib resistance have been reported: additional EGFR mutation (such as T790M), upregulation of bypass signals (such as ERBB2 and MET), and small-cell histologic transformation^26,27^. In addition to those, poor delivery or distribution of erlotinib are also possible mechanisms of resistance. To reveal whether poor distribution of erlotinib into tumours also results in erlotinib resistance, we investigated erlotinib distribution in erlotinib-refractory tumours prepared by long-term treatment with erlotinib. On the basis of MSI of erlotinib-refractory tumours it was considered that poor distribution of erlotinib into the tumours was not a major cause of resistance in this model. Thus, MSI provides valuable information with which to consider mechanisms of drug resistance.

This study suggested the distribution of small molecules within tumours is not necessarily affected by bevacizumab. In this case of erlotinib, other mechanisms involved in the combination therapy of erlotinib plus bevacizumab should be considered. Some reports have suggested that VEGF interacts directly with tumours^28–30^, and some kinds of lung cancer cells express VEGFR2^31,32^. Other studies have also reported that VEGF changes the tumour microenvironment^33,34^. The results of the current study imply that bevacizumab might affect the tumour cells directly or indirectly via inhibition of VEGF signals.

In this study, MALDI MSI was used to visualize erlotinib distribution. Various ionization techniques can be used for MSI, including secondary ion mass spectrometry, desorption electrospray ionization, and liquid extraction surface analysis^35–38^. Compared with these techniques, however, MALDI is able to handle a wider range of molecules and has a better balance of spatial resolution and sensitivity. As a consequence, MALDI was best suited to the aims of our study. Several other studies have also used MALDI MSI for erlotinib imaging^39–42^. A major advantage of MSI is that it can separate drugs and their metabolites, which is not able to be achieved by imaging techniques like autoradiography that involve labelling drugs. Owing to this advantage, erlotinib metabolites were able to be visualized separately from erlotinib. On the other hand, although MALDI MSI is sensitive enough for practical analysis of drug distribution, its sensitivity needs improvement. Greater sensitivity would enable us to analyse tumours at later time points in the erlotinib excretion phase when the concentration of erlotinib is much lower. Furthermore, sensitivity becomes a very important factor as we improve spatial resolution. Recently, a MALDI MSI system has been developed with the laser diameter drastically decreased to less than 1.5 µm^43^. However, since sensitivity is gained at the expense of spatial resolution (laser diameter), it is still difficult to apply MALDI MSI systems of this spatial resolution to drug distribution analysis. Even though MSI is still a developing technique, it is a promising technology that plays an important role in complementing auto-radiography to understand pharmacokinetics.

We have faced some limitations in this study. We measured erlotinib concentrations of adjacent tumour tissue sections as erlotinib concentration in tumour tissue sections analysed by MSI. Although it might have some influence on the concentration value, we thought it was the best way to estimate erlotinib concentration in imaged tissue. Because of damage to tumour tissue by MSI, we could not stain the tumour after MSI and could not overlap erlotinib distribution and details of parts of the tumour (microvessels, stroma and necrotic tissues, etc.). Since erlotinib was broadly distributed in the tumour in this study, we thought it was not necessarily needed. However, the technique to stain tumours after MSI is worth developing in the future for precise investigation of the location where erlotinib distributes.

We used the single cell line, B901L, as a model of EGFR-mutated NSCLC. Since the characteristics of tumours implanted into mice differ among cell lines, further studies covering several other cell lines would be informative to confirm our results. In addition, it is also worth testing models that do not use cell lines, such as genetically engineered mouse models and patient-derived xenograft models^44^. The current study was a fundamental study using a xenograft model bearing B901L tumours and is a first step in elucidating the mechanisms underlying the efficacy of combination therapy with erlotinib plus bevacizumab.

This study showed that bevacizumab in combination with erlotinib does not necessarily affect the distribution of erlotinib in EGFR-mutated NSCLC. The results of this study suggest that drug distribution profiles might possibly differ depending on the tumour type and the molecules administered in combination with bevacizumab. The mechanisms underlying the efficacy of combination therapy with antiangiogenic agents are still not fully elucidated, and accumulation of distribution data of various molecules in various tumours will be of great help in elucidating the mechanisms underlying the efficacy of combination therapy with antiangiogenic agents and small molecules. Multivariate approaches using MSI, such as were applied in this study, will contribute to elucidating this issue.

## Methods

### Chemicals

Bevacizumab was obtained from F. Hoffmann-La Roche (Basel, Switzerland). Human immunoglobulin G (human IgG) was purchased from MP Biomedicals (Santa Ana, CA, USA). Erlotinib for animal experiments was obtained from F. Hoffmann-La Roche. Erlotinib for LC-MS/MS analysis was purchased from Selleck Chemicals (Houston, TX, USA). Erlotinib D6 (stable isotope–labelled internal standard for erlotinib) was purchased from Toronto Research Chemicals (North York, ON, Canada). α-Cyano-4-hydroxycinnamic acid (α-CHCA) and 2,5-dihydroxybenzoic acid (DHB) were purchased from Sigma-Aldrich (St. Louis, MO, USA). HPLC grade trifluoroacetic acid (TFA), and LCMS grade methanol (for LC-MS/MS) were purchased from Kanto Chemical Co. (Tokyo, Japan). Formic acid, LCMS grade acetonitrile, and acetone were purchased from Wako Pure Chemical Industries (Osaka, Japan). The Qubit kit was purchased from Thermo Fisher Scientific.

### Tumour cell lines and culture conditions

The cell line B901L derived from lung cancer with EGFR deletion (E746–A750) was purchased from the Institute of Physical and Chemical Research (Saitama, Japan). This cell line was maintained in RPMI-1640 (Sigma-Aldrich) supplemented with 10% (v/v) foetal bovine serum (Bovogen Biologicals, Melbourne, Australia), 0.45% D-glucose (Sigma-Aldrich), 10 mM HEPES buffer (Sigma-Aldrich), and 1 mM Na-pyruvate (Thermo Fisher Scientific) at 37 °C under 5% CO_2_.

### Animal experiments

Male, 5-week-old BALB/c-nu/nu mice (CAnN.Cg-Foxn1 <nu> /CrlCrlj nu/nu) were obtained from Charles River Laboratories Japan (Kanagawa, Japan). Tumours were implanted by subcutaneous injection of B901L cells (5 × 10^6^ cells) into the mice. Tumours in the mice were grown for 14 to 18 days before receiving any treatment. The tumour-bearing mice underwent one of two treatment regimens (Table 1): a single dose of erlotinib (60 mg/kg) 5 days after a single dose of bevacizumab (5 mg/kg) (*n* = 15) or after a single dose of human IgG (5 mg/kg) as control (*n* = 15), or erlotinib (60 mg/kg) daily for 2 days (*n* = 5) or for 37 days (*n* = 5). Erlotinib was administered orally and bevacizumab was administered intraperitoneally. All animal experiments were reviewed and approved by the Institutional Animal Care and Use Committee at Chugai Pharmaceutical Co., Ltd., in accordance with the guidelines.

### Sample collection

Tumours and serum were collected from mice treated with erlotinib (60 mg/kg) following bevacizumab (5 mg/kg) or following human IgG (5 mg/kg) at 6 h, 12 h, 18 h after erlotinib treatment (*n* = 5 for each time point in each treatment group), and from mice treated with erlotinib (60 mg/kg) daily for 2 days or for 37 days at 3 h after last treatment (*n* = 5, each treatment group). Tumours that were not treated with any drugs were also prepared as erlotinib blank tumours. Tumours were frozen in liquid nitrogen immediately after collection.

### Immunohistochemistry and microvessel density analysis

CD31 in tissue sections was stained using a rat anti-mouse CD31 monoclonal antibody (BD Biosciences, San Jose, CA, USA) as described previously with some modifications^45^. In brief, rat anti-mouse CD31 monoclonal antibody bound to CD31 in tissue sections was detected by using Rat-HRP Polymer, 1-Step (Biocare Medical, Concord, CA, USA) containing XM Factor (Biocare Medical) followed by using DAB solution (Dako, Carpinteria, CA, USA).

Viable tumour areas were determined for each tumour tissue section by using a fluorescence microscope (BZ-X710; Keyence Corporation, Osaka, Japan). The CD31-stained area and the total viable cell area were calculated by using BZ-Analyzer (version 1.2.0.1; Keyence Corporation). Microvessel density was calculated by dividing the CD31-stained area by the total viable cell area.

### Measurement of erlotinib in serum and tumour tissue sections by LC-MS/MS

#### Sample pre-treatment

Serum samples (10 µL) were extracted with 90 µL of 5 ng/mL erlotinib D6 in methanol. Extracted samples were centrifuged at 12,000 × g at 4 °C. A 75 µL quantity of the supernatant was mixed with an equal volume of 0.2% formic acid. Calibration curve samples were prepared by spiking pooled mouse serum with erlotinib standard solutions. The calibration curve was linear over serum erlotinib concentrations ranging from 0.5 to 128 ng/mL. Samples were diluted up to 1000 fold, if needed.

Tissue sections were extracted with 75 µL of 5 ng/mL erlotinib D6 in methanol. The sample extracts were centrifuged at 12,000 × g at 4 °C. A 60 µL quantity of the supernatant was mixed with an equal volume of 0.2% formic acid. Calibration curve samples were prepared by spiking 5 ng/mL erlotinib D6 in methanol with erlotinib standard solutions. The calibration curve was linear over the concentration of 0.025 to 6.4 ng/mL erlotinib in sample extracts. Samples were diluted up to 4 fold, if needed.

#### LC-MS/MS analysis

Pre-treated samples were separated by liquid chromatography (Nexera × 2 series; Shimadzu) with an Xbridge C18 column (3.5 µm, 2.1 × 5.0 mm) (Waters, Milford, MA, USA) with temperature set at 40 °C. Mobile phases A and B were 0.1% aqueous solution of formic acid and 0.1% formic acid in acetonitrile, respectively; the flow rate was 0.2 mL/min and gradient elution was applied (Supplementary Table S2). Injection volume was 20 µL.

Erlotinib was quantified by using multiple reaction monitoring on a QTRAP 4500 LC-MS/MS system (AB Sciex, Framingham, MA, USA) in the electrospray ionization positive mode. The monitored transitions were 394 to 336 for erlotinib and 400 to 338.9 for erlotinib D6 (Supplementary Table 1). Data was processed using Analyst software (version 1.6.1, AB Sciex). Erlotinib concentration in tumour tissues was calculated by dividing the quantity of erlotinib in the tissue section by the quantity of protein in the adjacent tissue section.

#### Measurement of protein amounts in tumour tissue sections

Tumour tissue sections were homogenized with 40 µL of ultrapure water and the protein concentrations were measured by using the Qubit fluorometer (Thermo Fisher Scientific) according to the manufacture’s manual. The quantity of protein in the tumour section was calculated from the concentration.

### MSI

#### Sectioning

Tumour tissues were sliced into 8 µm sections using a cryomicrotome (CM1950; Leica Microsystems K.K., Tokyo, Japan). Three consecutive tissue sections were prepared for MSI analysis. The first section was mounted on an ITO-coated glass slide (Bruker, Billerica, MA, USA) for analysis using iMScope or mounted on a glass slide (Matsunami Glass Ind., Osaka, Japan) for analysis using Q Exactive; the second section was placed in a polypropylene (PP) tube for quantitation of erlotinib by LC-MS/MS; and the third section was placed in a PP tube and used for protein quantitation. Tissue sections were stored at −80 °C until use.

#### Matrix application

Optical images of tumour tissue sections were acquired before matrix application using iMScope and the fluorescence microscope BZ-X710 for analysis using iMscope and Q Exactive respectively. For analysis using iMScope, α-CHCA was vapour-deposited at 250 °C onto the surface of the tumour tissue sections as previously described^19^, and then 50% acetonitrile containing 7 mg/mL α-CHCA, 1 µg/mL erlotinib D6, and 0.2% TFA was applied by using the ImagePrep matrix deposition device (Bruker) according to manufacturer’s standard method for α-CHCA. In ImagePrep system, number of spray cycles were controlled by thickness on ITO glass measured by the sensor. For analysis using Q Exactive, 50% acetone containing 30 mg/mL DHB, 150 ng/mL erlotinib D6 was applied to tumour tissue sections by using ultrafine sprayer system (SMALDIprep; TransMIT) (Supplementary Table S3).

#### MALDI-MSI

Erlotinib in tumour tissue sections was visualized by using 2 different MALDI MSI systems, iMScope (Shimadzu) and Q Exactive (Thermo Fisher Scientific), with AP-SMALDI (TransMIT) as the ion source. One tissue was analysed at a time. The mass resolving powers of the instruments were 10,000 at *m*/*z* 1000 for iMScope and 140,000 at *m*/*z* 400 for Q Exactive. For iMScope, spectra were acquired in positive mode by tandem MS analysis at a spatial resolution of 80 µm for whole-section imaging or 40 µm for partial-section imaging. For erlotinib analysis, the *m*/*z* range of the precursor ion was 394.1 ± 1.5 and the acquired *m*/*z* range of the product ion was 100 to 390. For erlotinib D6 analysis, the *m*/*z* range of the precursor ion was 400.1 ± 1.5 and the acquired *m*/*z* range of the product ion was 100 to 410. For heme b analysis, the *m*/*z* range of the precursor ion was 616.2 ± 1.5 and the acquired *m*/*z* range of the product ion was 500 to 650. For analysis of erlotinib D6 in whole-section imaging, the laser shots were displaced 40 µm in both the *x* and *y* directions from the burned spots used to measure erlotinib. For analysis of erlotinib in the partial-section imaging, the laser shots were displaced 20 µm in both the *x* and *y* directions from the burned spots used to measure heme b. For Q Exactive, spectra were acquired in positive mode at a spatial resolution of 30 µm. For analysis of erlotinib and erlotinib metabolites (M14 and M13), all analytes were measured simultaneously. Acquired *m*/*z* range was 378 to 403.

Analyte distributions were processed and visualized using Imaging MS Solutions software (version 1.11; Shimadzu) for iMScope and Mirion software (version 3.1.64.4; TransMIT) for Q Exactive. Intensities were expressed on a colour gradient scale, and the maximum value of the intensity scale was adjusted for each image. For iMScope, *m*/*z* 336.13 ± 0.05, 339.15 ± 0.05, and 557.17 ± 0.05 were used to visualize erlotinib, erlotinib D6, and heme b, respectively (Supplementary Table S1). Normalization by erlotinib D6 as internal standard was not conducted since internal standard normalization had little impact on the distribution (data not shown). For Q Exactive, *m*/*z* 394.1761 ± 3 ppm, and 380.1605 ± 3 ppm were used to visualize erlotinib and metabolites (M14 or M13), respectively (Supplementary Table S1).

### Statistical analysis

Statistical analysis was conducted using JMP software (version 11.2.1; SAS Institute Inc., Cary, NC, USA). Mean ± SD was used for graph building unless otherwise mentioned. Significances were tested using 2-sided Student’s t-test (P < 0.05).

## Electronic supplementary material

Supplementary Information
