# Supplementary material for: MALDI mass spectrometry imaging of erlotinib administered in combination with bevacizumab in xenograft mice bearing B901L, EGFR-mutated NSCLC cells
Source: Sci Rep. 2017 Dec 1;7:16763. doi: 10.1038/s41598-017-17211-6 (PMC5711937; doi:10.1038/s41598-017-17211-6)
Supplement: Supplementary file 1 — Supplementary Information [file 41598_2017_17211_MOESM1_ESM.pdf]

Supplementary Information

MALDI mass spectrometry imaging of erlotinib administered in combination with bevacizumab in xenograft mice bearing B901L, EGFR-mutated NSCLC cells

Masanobu Nishidate[1,4,5] Kaname Yamamoto[2], Chinami Masuda[2], Hiroaki Aikawa[3], Mitsuhiro Hayashi[3,4], Takehiko Kawanishi[1], Akinobu Hamada[*,3,4,5]

Affiliation information

[1]Translational Clinical Research Science & Strategy Dept., Chugai Pharmaceutical Co., Ltd., 200 Kajiwara, Kamakura, Kanagawa 247-8530, Japan.

[2]Product Research Dept., Chugai Pharmaceutical Co., Ltd., 200 Kajiwara, Kamakura, Kanagawa 247-8530, Japan.

[3]Division of Clinical Pharmacology and Translational Research, Exploratory Oncology Research and Clinical Trial Center, National Cancer Center, 5-1-1 Tsukiji, Chuo-ku, Tokyo 104-0045, Japan.

[4]Department of Molecular Pharmacology, National Cancer Center Research Institute, 5-1-1 Tsukiji, Chuo-ku, Tokyo 104-0045, Japan.

[5]Department of Medical Oncology and Translational Research, Graduate school of Medical Sciences, Kumamoto University, 1-1-1 Honjo, Chuo-ku, Kumamoto 860-8556, Japan.

Correspondence and requests for materials should be addressed to A.H. (email: akhamad@ncc.go.jp)

Supplemental Methods

Confirmation of spectra of erlotinib reference material

For analysis using iMScope, 0.001 mg/mL of erlotinib in 50% methanol was spotted on an ITO-coated glass slide (Bruker, Billerica, MA, USA), α-CHCA was vapour-deposited at 250˚C onto the surface of the spot as described in the methods of this paper, and then 50% acetonitrile containing 7 mg/mL α-CHCA and 0.2% TFA was spotted on the spot of erlotinib. For analysis using Q Exactive, 1 mg/mL of erlotinib in 50% methanol was spotted on ITO-coated glass slide (Matsunami Glass Ind., Osaka, Japan) and 50% acetone containing 30 mg/mL DHB was applied to the spot by using ultrafine sprayer system (SMALDIprep; TransMIT). MS and MSMS spectra of the spots were acquired according to the methods described in this paper.

Normalization by erlotinib D6

Erlotinib distributions were processed and visualized using BioMap (version 3.8.0.4; Novartis Institutes for BioMedical Research, Basel, Switzerland). Intensity of erlotinib (m/z 336.13 $\pm$ 0.05) was divided by corresponding intensity of erlotinib D6 (339.15 $\pm$ 0.05).

Supplemental Table 1. m/z list used in each MS instrument.

Analytes	QTRAP4500 (precursor>product)	iMScope (precursor>product)	Q-Exactive
Erlotinib	394>336	394.1>336.13	394.1761
Erlotinib D6	400>338.9	400.1>339.15	-
M14, M13	-	-	380.1605
Heme b	-	616.2>557.17	-

Supplemental Table 2. Gradient conditions of LC

Time (min)	Flow rate (mL/min)	Mobile phase A	Mobile phase B
0.00	0.2	80	20
4.00	0.2	35	65
4.10	0.2	10	90
5.10	0.2	10	90
5.20	0.2	80	20
8.00	0.2	80	20

Mobile phase A: 0.1% aqueous solution of formic acid
Mobile phase B: 0.1% formic acid in acetonitrile

Supplemental Table 3. SMALDIprep condition

Parameters	Setting
Gas	Nitrogen
Gas flow rate	5 L/min
Matrix flow rate	5 μL/min
Rotation speed	350 rpm
Sprayer Hight	56.3 mm

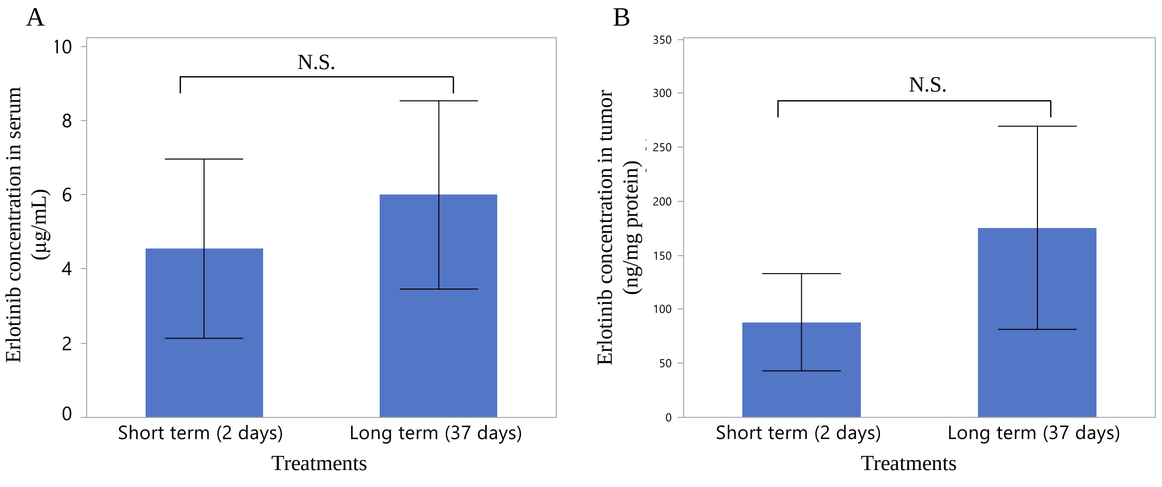

Supplementary Figure S1. Erlotinib concentrations in serum and tumours in mice treated with erlotinib daily for 2 days or for 37 days. Samples were collected at 3 h after the last treatment. Erlotinib concentrations in serum (**A**) and tumours (**B**) were measured using LC-MS/MS. Mean ± SD are shown.

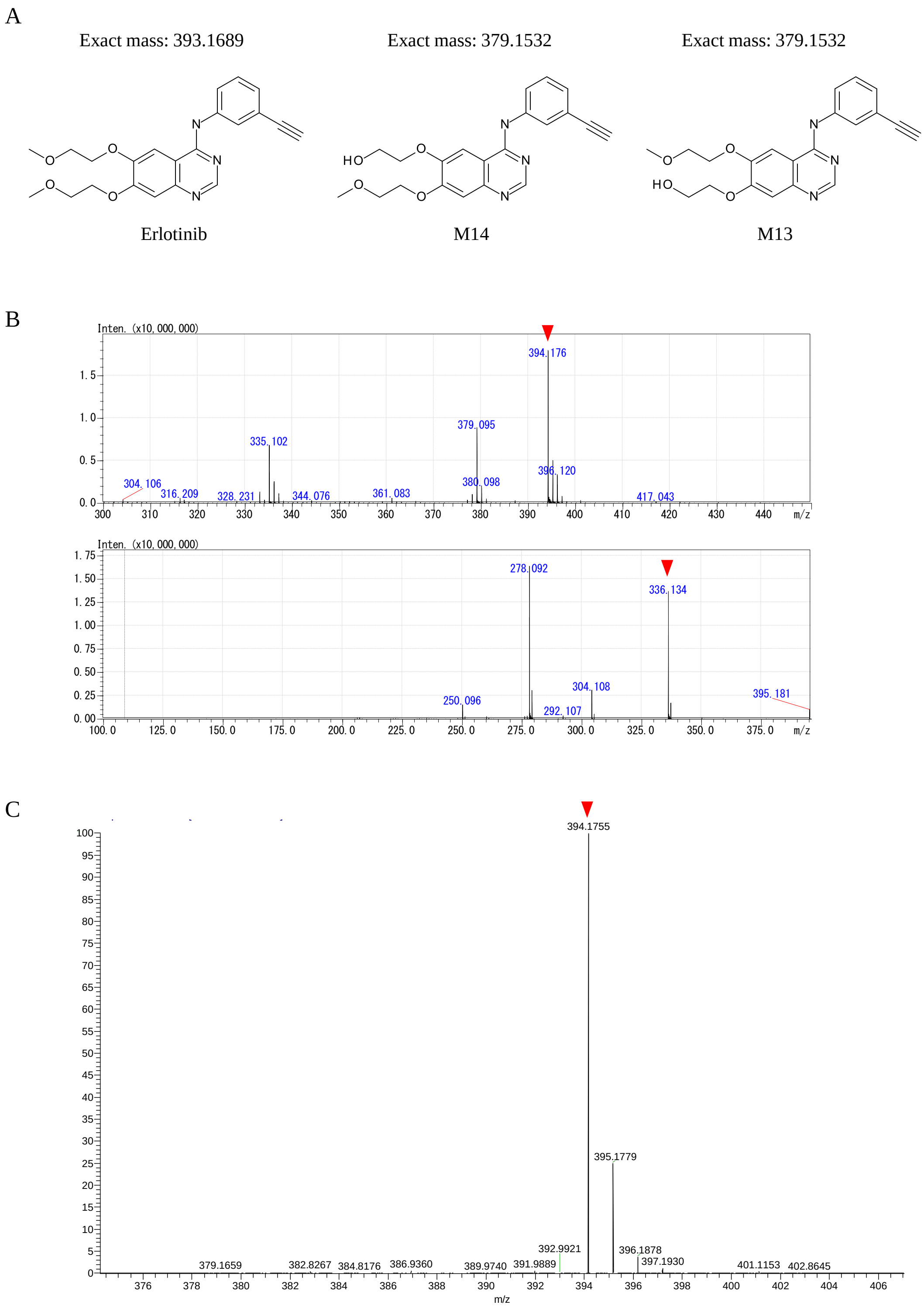

Supplementary Figure S2. Chemical formula and spectra of erlotinib. (**A**) Chemical formulae of erlotinib and its metabolites. (B) MS (upper) and MSMS (lower) spectra of erlotinib reference material produced by iMScope. (C) MS spectra of erlotinib reference material produced by Q Exactive. Arrowheads indicate monitored ion of erlotinib.

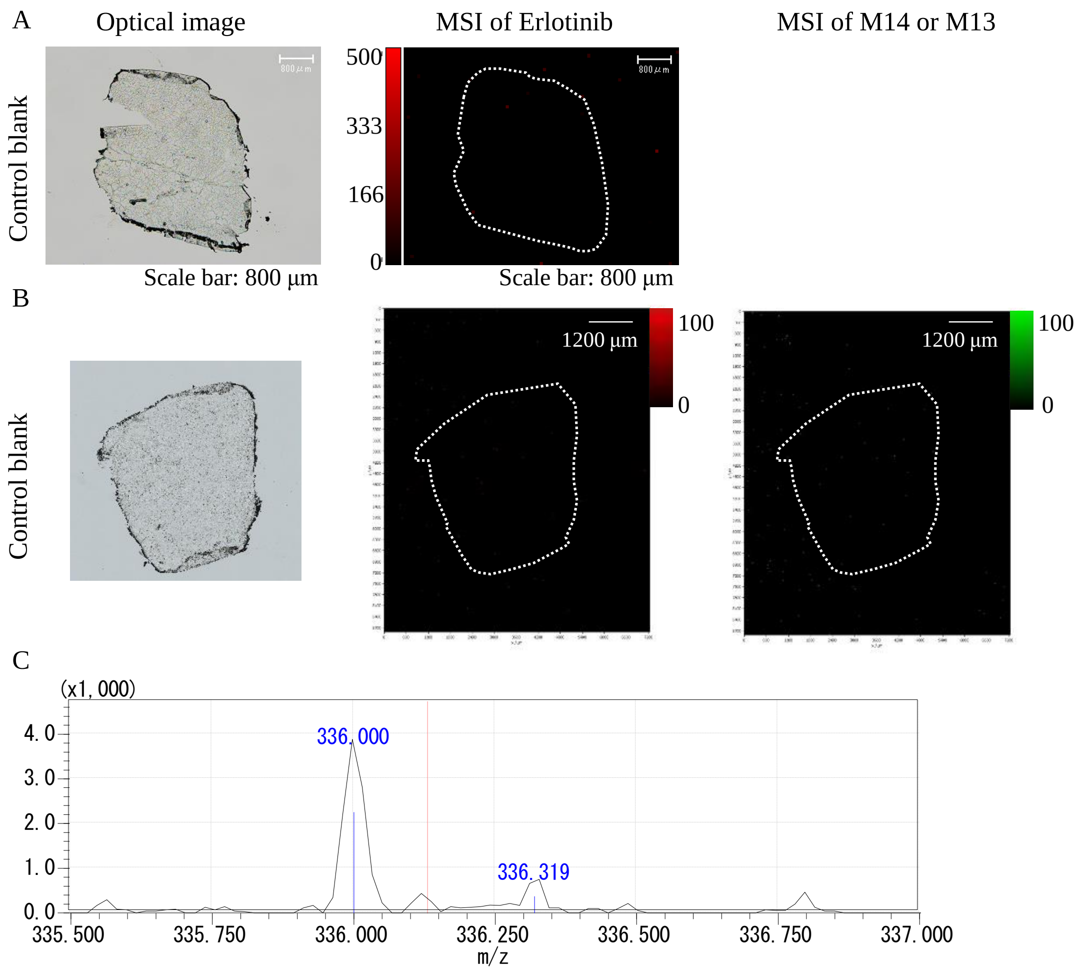

Supplementary Figure S3. MSI of blank tumours. Images of tumours without any treatment produced by iMScope (**A**) and Q Exactive (B). Optical images were acquired just before MSI, and were the same sections as the imaged sections. The maximum value on the intensity scale was adjusted for each image. Erlotinib signals are indicated by red and intensity of metabolites is indicated by green. (C) Mean spectra in the brank tumour analyzed by iMScope. Red line indicates m/z 336.13. No non-specific signals were observed.

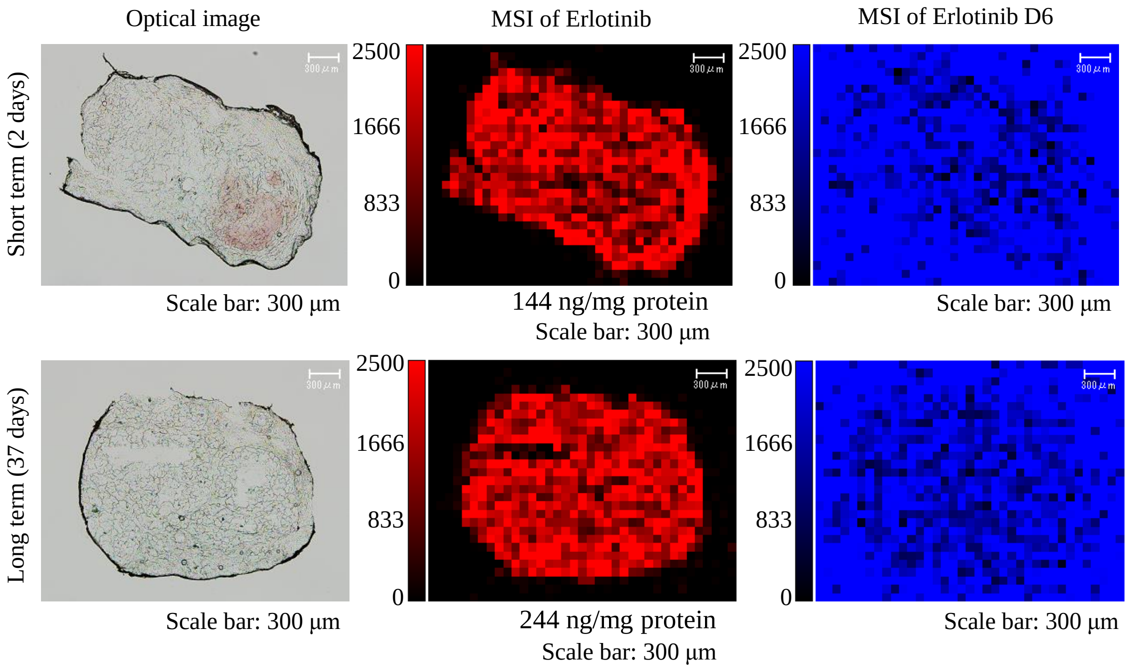

Supplementary Figure S4. Erlotinib distribution in tumours treated with erlotinib daily for 2 days (upper panels) or 37 days (lower panels). Tumours were collected at 3 h after last treatment. Concentrations of erlotinib in an adjacent tissue section was indicated below erlotinib imaging (centre). Erlotinib D6 deposited with matrix was imaged to grasp ionization trends on tissues. Data was obtained using iMScope with 80 μm spatial resolution. The maximum value on the intensity scale was adjusted for each image. Erlotinib signals are indicated by red and erlotinib D6 intensity is indicated by blue.

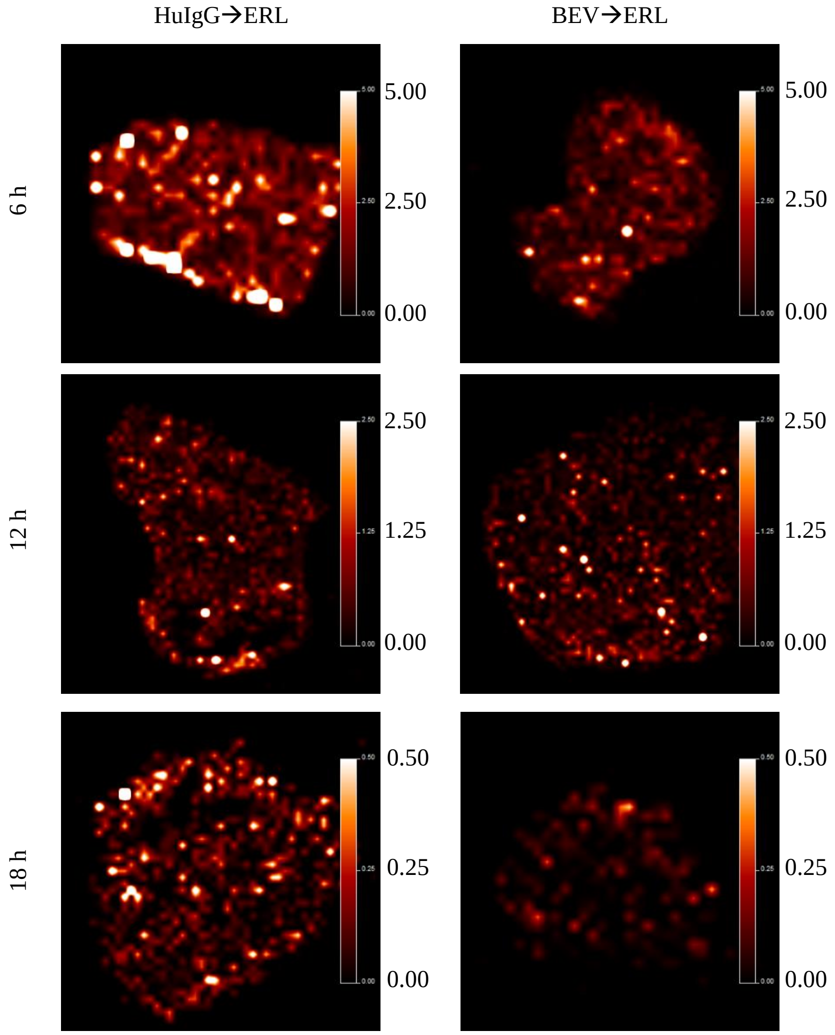

Supplementary Figure S5. Erlotinib distribution normalized by erlotinib D6 in tumours from mice treated with erlotinib (ERL) following human IgG (HuIgG, left panels) or bevacizumab (BEV, right panels). The maximum value on the intensity (ERL/ERL D6) scale was adjusted for each time point.
